# Controversies and Advances in the Personalized Surgical Treatment of Cervical Cancer

**DOI:** 10.3390/jpm14060606

**Published:** 2024-06-06

**Authors:** Vasilios Pergialiotis, Ioannis Rodolakis, Alexandros Rodolakis, Nikolaos Thomakos

**Affiliations:** First Department of Obstetrics and Gynecology, Division of Gynecologic Oncology, “Alexandra” General Hospital, National and Kapodistrian University of Athens, 15703 Athens, Greece; john_rodolakis@hotmail.com (I.R.); a.rodolaki@gmail.com (A.R.); nthomakos@med.uoa.gr (N.T.)

**Keywords:** cervical cancer, personalized treatment, survival outcomes, novel approaches

## Abstract

Cervical cancer represents a global health issue as it is mostly encountered in women of reproductive age, while at the same time, survival outcomes seem to have remained constant during the last two decades. The need to implement fertility-sparing strategies as well as to decrease the morbidity that accompanies radical treatment has been extensively studied. During the last decade, several randomized clinical trials have been released, resulting in significant advances in the surgical treatment of early-stage disease. At the same time, evidence about the surgical treatment of advanced-stage disease as well as recurrent disease has gradually appeared and seems to be promising, thus leading the point forward towards personalized medicine that will remove the surgical barriers that seem concrete in our era. Nevertheless, the discrepancies in perioperative morbidity and survival outcomes that were observed among published studies raise several questions. In the present article, we chose to review the gray fields in the surgical treatment of early-stage and advanced-stage cervical cancer. Studies that are based on strong evidence that support current clinical practice are compared to smaller cohorts that present novel data that may form the basis for future research, and issues that remain poorly explored are discussed in an effort to help establish a consensus for future research development.

## 1. Introduction

Cervical cancer is the fourth most common gynecologic malignancy, with a global estimate of approximately 600,000 cases in 2020 and 341,000 deaths according to the Global Cancer Observatory (GLOBOCAN) [1]. With the development of vaccines, the prevalence of cervical cancer has gradually declined; however, in countries with insufficient resources, the problem persists [2]. Therefore, while its prevalence has markedly declined in countries with a high Human Development Index, countries with insufficient and opportunistic health care services have six times higher mortality rates, indicating the extent of the problem [1]. It is estimated that approximately 60% of patients will seek help at the advanced stage, therefore precluding surgical treatment as a primary option [3].

The newly released European Society of Gynecologic Oncology (ESGO) recommendations have extended the limits of surgery by increasing the number of cases requiring less radical procedures and by re-instituting once more minimally invasive surgery as an option for small-volume tumors [4]. Several randomized trials have been released in the last decade that took surgical treatment of early-stage disease several steps forward, whereas evidence about the surgical treatment of advanced-stage disease as well as recurrent disease has gradually appeared and seems to be promising, thus leading the point forward towards personalized medicine that will remove the surgical barriers that seem concrete in our era.

In the present article, we chose to review the gray fields in the surgical treatment of early-stage and advanced-stage cervical cancer. Studies that are based on strong evidence that support current clinical practice are compared to smaller cohorts that present novel data that may form the basis for future research, and issues that remain poorly explored are discussed in an effort to help establish a consensus for future research development. 

## 2. Early-Stage Disease Challenges

### 2.1. Surgical Radicality

Patients with early-stage disease pose a clinical challenge as their management is dictated by the evaluation of several factors that determine not only the extent of the surgical procedure but also the need for adjuvant therapy. Current recommendations for cervical cancer treatment suggest that tumors <0.5 cm detected at a simple hysterectomy specimen do not require further treatment in the absence of lymphovascular space involvement (LVSI); however, cases with LVSI should be offered comprehensive lymphadenectomy [4]. Considering this, it is reasonable to assume that in these cases a simple hysterectomy might suffice and could be instituted in current clinical practice. In a retrospective chart review of 1530 women with stage Ia2 cervical cancer and 3931 women with stage IB1 cervical cancer that was retrieved from the National Cancer Database, researchers observed that after implementing propensity score matching, the radicality of the procedure did not influence survival rates of patients with Ia2 (FIGO 2018 classification) disease [5]. Patients with stage IB1 disease, on the other hand, had a 55% increase in the risk of death, and 5-year survival rates were 95.3% for radical hysterectomy compared to 92.4% for simple hysterectomy. The impact of less extensive surgery for patients with FIGO stage Ia2 has since been investigated by other researchers with similar findings, thus denoting the possibility of de-escalating the radicality of the procedure and potentially abandoning lymphadenectomy for these patients [6]. To help establish a robust baseline that could have an impact on clinical practice, the LESSER trial was designed as a phase II non-inferiority study [7]. The trial, which recruited 40 patients in a 1:1 allocation ratio, investigated the impact of non-radical surgery in patients with Ia1-Ib1 disease (tumor diameter <2 cm), and its findings suggested that the 5-year overall survival rates of patients offered simple hysterectomy may be similar to those of patients offered modified radical hysterectomy (90% vs. 91%, respectively). However, due to the relatively small number of recruited patients, the impact of intermediate risk factors (as analyzed below) on survival rates was not assessed. The preliminary results of the highly anticipated SHAPE trial (NCT01658930) that investigates the difference in survival rates of women undergoing modified radical versus simple hysterectomy with pelvic lymphadenectomy seem to be extremely favorable, as pelvic and extrapelvic recurrences as well as the overall survival of patients seem to be comparable in the two groups [8,9]. The trial also indicates a detrimental impact on quality-of-life outcomes, which were by far superior in the simple hysterectomy group. Unfortunately, subgroup analysis of patients assigned to stage Ia2 compared to those assigned to stage Ib1 has not been made available yet.

Considering this, it is reasonable to assume that hysterectomy is still not an option for tumors with a stromal invasion >0.5 cm [10]. However, even if this will be the case in the future, the need to determine the extent of radical surgery still remains, as this is directly related to perioperative and long-term postoperative complications. A recent multi-institutional study tailored the survival benefit of the extent of the procedure in patients with FIGO 2009 stages Ib1 and IIa1 [11]. The study involved 1257 patients that were offered radical hysterectomy by laparotomy, and it was observed that the extent of radicality of the procedure had a significant impact on disease-free survival (DFS) of patients with tumors with a diameter that ranged between 2 and 4 cm (current FIGO staging Ib2) (83.1% for nerve-sparing cases vs. 90.3% for non-nerve-sparing cases; *p* = 0.016).

### 2.2. Impact of Positive Lymph Nodes on Decision to Abandon Hysterectomy

Estimation of the lymphatic status with sentinel lymph node sampling and frozen section pathology is of particular importance in the presence of enlarged suspicious lymph nodes and is considered in the presence of non-suspicious lymph nodes as an option by the newest ESGO guidelines [4]. Alternatively, a two-step approach is considered, with minimally invasive lymphadenectomy taking place first and surgery being completed following final pathology. The reason is that the procedure is discontinued/aborted in the event of identified macro- or metastatic disease, whereas the presence of isolated tumor cells is still an arguable factor for the use of adjuvant radiotherapy. The results are based on earlier studies that support discontinuation of the procedure on the basis of a potential marginal improvement in the overall survival of patients treated only with radiotherapy [12,13,14,15]. It is also considered that the combination of radical surgery with radiotherapy may increase the risk of complications, including fistula formation. However, one study denoted the possibility of improved local control and increased progression-free survival in carefully selected patients with favorable prognostic features [16]. Another large cohort study that involved 121 patients of whom 89 completed the procedure and received adjuvant chemoradiotherapy and 32 received standard treatment with chemoradiotherapy following discontinuation of the operation also suggested a significant decrease in relapses among those that completed the operation (15.7% vs. 31.2% in patients for whom hysterectomy was abandoned) [17]. The most prominent decrease was in pelvic recurrences (*p* = 0.014), as distant metastases remained unaffected. Similarly, the time to recurrence significantly differed (18 vs. 11 months, *p* = 0.011). The 5-year disease-free survival was considerably higher in the radical hysterectomy group (*log-rank* = 0.034); however, the overall survival was not significantly different (*log-rank* = 0.298). Taking this information into account, the ABRAX (ABandoning RAdical hysterectomy in cerviX cancer) international, multicenter, retrospective cohort study was undertaken to evaluate the actual impact of surgery in the presence of positive lymph nodes. The study involved 515 cases from 51 institutions [18] at a median follow-up of 58 months, during which 133 patients experienced disease relapse and 100 died. Pelvic recurrences were noted in 59 patients. Researchers reported that the risk of recurrence was similar between the two groups (HR 1.154, 95% CI 0.799–1.666) and also noted that pelvic recurrences also did not differ (HR 0.836, 95% CI 0.458, 1.523). Consequently, the risk of death was comparable (HR 1.064, 95% CI 0.690, 1.641). Of note, treatment-related adverse events after the first month following the operation were higher in the group for whom hysterectomy was abandoned (16% vs. 9%). Taking this information into consideration, it must be considered that until further information becomes available, completion of the procedure should be avoided in patients with lymph node metastases as it does not provide a survival benefit. It should be noted that, although both macro- and micrometastases were considered in the inclusion of patients, subgroup analysis considering this comparison was not performed to evaluate if there is a potential difference in survival outcomes in the presence of micrometastatic disease. One could assume, therefore, that further investigation might reveal different outcomes among those patients, as their survival might considerably differ, as well as their need for adjuvant treatment [19,20,21]. 

### 2.3. Factors Determining the Need for Adjuvant Treatment

Several issues arise when considering the possibility of adjuvant therapy in patients offered surgery for cervical cancer, including the presence of intermediate risk factors. These involve the presence of a combination of the following risk factors: presence of LVSI, >1/3 stromal invasion and tumor size >4 cm (as indicated by clinical examination) [22]. Close or positive surgical margins and tumor histology (adenocarcinoma subtype) have also been addressed by the National Comprehensive Cancer Network (NCCN) and are considered in current clinical practice as well. It should be noted, however, that despite advances in modern pathology and in surgical, clinical and radiation oncology, the management of these patients has remained the same for the last two decades, thus posing the question of if these criteria need to be updated [23]. 

Currently, two ongoing clinical trials are being conducted to determine the impact of intermediate risk factors on survival rates of patients with early-stage disease. The first of these (GOG-0263) includes patients with stage I-IIA disease and assesses the impact of chemoradiotherapy on disease-free survival and overall survival of patients with LVSI and (i) deep third penetration or (ii) middle third penetration with a clinical tumor diameter >2 cm, or (iii) superficial third penetration with a clinical tumor diameter >5 cm [24]. Patients with negative LVSI and middle or deep third penetration with a tumor diameter >4 cm will also be assessed. The estimated primary completion date is in April 2024. The second study (CERVANTES) recruits patients with FIGO stage Ib1-IIa disease and assesses the presence of (i) tumor diameter ≥4 cm, (ii) a tumor >2 cm <4 cm with concurrent lymphovascular space invasion, (iii) a tumor >2 cm <4 cm in the presence of tumor-free distance <3 mm, or (iv) a tumor >2 cm <4 cm accompanied by deep stromal invasion (>2/3) [25]. Its primary results are expected to be released after 2029. 

### 2.4. Minimally Invasive Surgery

The initial benefits of minimally invasive radical hysterectomy were completely downgraded by the LACC trial (NCT00614211) that was discontinued earlier than anticipated due to the increased relapse rate that was observed in the minimally invasive group, which was accompanied by a significant risk of death from the disease (HR 6.00, 95% CI, 1.77–20.30) [26]. The considerable aftermath of the study had direct implications in current clinical practice, with the use of minimally invasive procedures decreasing from 65% to 30% of cases according to the findings of a retrospective multi-institutional study [27]. Several risk factors have been proposed as potential causes that drove the poor survival outcomes, including the size of the lesion (using a 2 cm cut-off), the use of a uterine manipulator, the impact of prior conization and the surgical expertise, which was, however, evaluated prior to enrollment in the LACC trial. It should be noted, however, that the safety of the procedure seems to be problematic even among cases with small lesions (<2 cm), as a recent systematic review denoted decreased progression-free (HR 1.68, 95% CI 1.20–2.36) and overall survival (HR 1.64, 95% CI 1.00–2.68) compared to standard laparotomy even after control of potential confounders [28].

Considering this, it became apparent that the use of protective measures, by means of vaginal stump closure prior to excision of the specimen, might be the solution to the problem. A retrospective study that was released shortly after the LACC trial revealed that the combination of laparoscopic radical hysterectomy with transvaginal cuff closure and avoidance of use of a uterine manipulator provides excellent oncologic outcomes [29]. Similar results were obtained for smaller cohorts that also used a non-touch technique, indicating that this factor should be considered in future clinical trials [30,31]. 

In the aftermath of the LACC trial, the actual role of robotic surgery remains questionable, largely due to the paucity of prospective data. It should be noted that at least one large nationwide population retrospective study revealed that for patients with stage IA1-IB disease, robotic radical hysterectomy should be considered feasible, with minimal impact on recurrence and long-term overall survival, indicating the necessity for further studies in the field [32]. Similar results were also observed in other multi-institution cohorts synchronous to the LACC trial [33]. Considering that robotic surgery is considered a more sophisticated surgical approach due to the increment in the degrees of freedom that it offers to the operator compared to standard laparoscopy, one could assume that the minimal handling of tissues could also benefit patients, although this remains to be proven in the future. The ROCC trial (Robotic Versus Open Hysterectomy Surgery in Cervix Cancer)—NCT04831580 is highly anticipated in this regard; however, its results will be considered final in the second semester of 2029 [34].

An issue that has not been investigated appropriately also lies in the unexpected identification of high-risk factors during the procedure. Recently, a Korean study revealed that the oncological safety of minimally invasive procedures was comparable to that of open surgery even in the presence of parametrial invasion and/or lymph node metastases on the final pathology report, provided that adjuvant therapy was used [35]. 

Considering all these data, it becomes apparent that further research is required in this field as the reduction in minimally invasive procedures has resulted in a direct increase in postoperative morbidity [27].

### 2.5. Tumor Reduction before Radical Surgery for Early-Stage Disease

#### 2.5.1. Conization Prior to Radical Surgery

The importance of cervical conization prior to definitive surgical treatment with radical hysterectomy has been discussed by several researchers during the last decade. The results of the SUCCOR multi-institutional retrospective cohort indicated that patients with a tumor diameter <4 cm (FIGO 2009 stage IB1) had considerably better outcomes when conization was performed prior to definitive surgery [36]. The importance of the finding was underlined by a significant decrease in relapse rates (65% reduction). In another retrospective study that was conducted in Korea and which involved 1799 patients of whom 291 underwent preoperative conization, researchers denoted that this finding may be true, as the multivariate analysis showed that conization was associated with a 55% reduction in recurrence rates (HR 0.65, 95% CI 0.34–1.02) and a 41% reduction in death rates (HR 0.59, 95% CI 0.34–1.02), both of them achieving a marginal statistical significance [37]. It should be noted that the large sample size permitted the use of propensity score matching, hence limiting the possibility of bias that arises from several confounding factors, including stage, grade of differentiation, patient age, type of procedure (radicality and use of minimally invasive techniques), presence of lymphovascular space involvement and parametrial involvement and positive resection margins. 

To date, the use of cervical conization prior to definitive surgical treatment has not been adopted, and it would be of particular interest to investigate it especially in patients with stage Ib2 disease as well as in those with tumors <2 cm that have intermediate factors of disease recurrence. Women undergoing radical trachelectomy as part of fertility-sparing surgery for tumors with a diameter 2–4 cm (stage Ib2) might also benefit from the addition of cervical conization, and a direct comparison of survival outcomes to those achieved by the use of neoadjuvant chemotherapy prior to cervical trachelectomy might be of particular importance. 

#### 2.5.2. Neoadjuvant Chemotherapy Prior to Fertility-Sparing Treatment

The concept of using neoadjuvant chemotherapy (NACT) prior to fertility-sparing treatment has gained significant ground during the last 5 years as a means that can help women with larger tumors achieve appropriate tumor reduction that will help achieve optimal local control of the disease. An issue that arose was related to the actual estimation of lymphatic disease at the time of surgery, and the latest update of the ESGO guidelines suggests that minimally invasive lymph node assessment is essential prior to the onset of neoadjuvant chemotherapy and must include pelvic and presacral lymph nodes [4]. The concept of this method was firstly reported in 2006 by Plante et al. [38] and has since gained significant ground as it presents an opportunity for women with bulky cervical disease to retain their fertility. Its use has been mainly adopted as a means that will help reduce the extent of the procedure, which will concurrently help achieve better pregnancy rates. A recent systematic review that summarized evidence from 352 patients reported indeed that the use of NACT accompanied by less radical surgery considerably increased pregnancy rates and birth rates compared to standard fertility-sparing treatment with radical hysterectomy [39]. Survival rates were not, however, reported in this study. Morice et al. summarized the evidence by systematically searching for studies published in international databases and retrieved outcomes from 275 patients that revealed considerably higher risks of recurrence following neoadjuvant chemotherapy compared to radical trachelectomy (13.2% vs. 4.8%) [40]. Similar results were published in a recent retrospective study that revealed, however, that the rates of recurrence were increased mainly in patients that achieved a <50% tumor reduction following NACT [41].

Considering these findings, physicians must counterbalance patients’ needs and always mention the higher risks of disease recurrence, especially in the absence of adequate response to NACT. Estimation of the actual impact of intermediate factors of disease recurrence could be of some use; however, this has been poorly investigated by researchers as the majority of published studies are based on relatively small sample sizes that do not favor subgrouping of patients.

## 3. Advanced-Stage Disease Challenges

Traditionally, advanced-stage disease is treated with concurrent chemoradiotherapy according to the latest FIGO and NCCN guidelines [4,22]. Recommendations include the use of external beam radiotherapy at a dose of 45 Gy/25 fractions or 46 Gy/23 fractions using IMRT (intensity-modulated radiotherapy) with concomitant use of cisplatin in weekly doses. Despite the significant survival benefits of the technique, surgeons have explored the potential benefit of tumor reduction prior to or even after the completion of radiotherapy. The results of these studies are discussed below. 

### 3.1. Debulking Surgery Prior to Chemoradiotherapy

The concept of surgical removal of bulky disease has been investigated prior to the onset of chemoradiotherapy as well. In a retrospective analysis based on patients from the Surveillance, Epidemiology and End Results (SEER) database, researchers observed that patients with stage IV disease that underwent removal of the primary and/or metastatic disease had considerably lower odds of death (HR 0.47, 95% CI 0.29–0.76) [42]. The 3-year and 5-year overall survival rates for the operated group were 48.4% and 41.5%, respectively, compared to 25.3% and 20.5% for those that received chemoradiotherapy only. Stratified analysis indicated that patients with small-volume disease in the primary site (<4 cm) as well as those that did not have visceral metastases received the greatest benefit from the operation. Considering, however, the morbidity that accompanies radical surgery and keeping in mind the results of the SUCCOR study in patients with early-stage disease, it would be worthwhile to investigate the impact of local control by implementing tumor reduction with cervical conization prior to the onset of chemoradiotherapy, particularly in patients with localized bulky disease (stages Ib3 and IIa2). To date, such studies are lacking and would be of particular interest. 

#### Lymphadenectomy Prior to Chemoradiotherapy

Lymphatic disease is associated with considerably worse survival outcomes and is treated with concurrent chemoradiotherapy that covers the paraaortic lymph nodes as well. Treatment of bulky lymph nodes in locally advanced cervical cancer does not seem to be accompanied by significant differences in cervical outcomes [43]. However, it is also known that bulky lymph nodes require high-dose boosted external beam radiotherapy (EBRT) to become sterilized. To date, the available evidence concerning the impact of debulking surgery on bulky lymphatic disease provides heterogeneous results, with some studies favoring the technique [44,45] and others providing evidence against it [43,46,47]. A prospective phase III randomized trial (CQGOG0103) will help provide more information on this group of patients; however, its results are not anticipated within this decade as the enrollment will last four years and the follow-up will be completed at five years [48].

### 3.2. Completion Hysterectomy

Completion hysterectomy has been investigated in locally advanced cervical cancer in the last two decades. The rationale behind this approach is based on the hypothesis of regional control of the disease. To date, most of the published evidence relies on retrospective studies that compared patients receiving radiotherapy or chemoradiotherapy to patients that received either form of those treatments complemented by adjuvant hysterectomy. A recent network meta-analysis showed that completion hysterectomy did not significantly affect the survival rates of those patients [49]. It should be noted, however, that the investigated studies were particularly heterogeneous, as the radicality rate of the reported procedures varied considerably, as well as the stage of the disease of the included patients. Therefore, it remains unknown whether selected cases with locally advanced disease that is associated with improved survival, including cases with stage IB3 and IIA2 disease, might benefit from the use of adjuvant hysterectomy following systemic chemoradiotherapy. To help establish this knowledge, the researchers designed a multicenter prospective randomized trial (C-CRAL trial) which will enroll 402 patients over a period of 3 years [50]. 

## 4. Follow–Up Prophylactic Vaccination

The role of prophylactic HPV vaccination as an adjuvant prophylaxis following surgical treatment of preinvasive disease has been documented by several researchers [51]. The rationale behind this observation relies on the reduction in the percentage of HPV-infected cells following cervical conization, which significantly limits the possibility of disease recurrence [52]. A recent meta-analysis indicated, however, that this effect might be prone to small study effects, including inherent bias of retrospective studies, as the authors denoted that when they considered only the results of studies with the least possibility of bias, the result was not consistent with a beneficial effect [53]. The actual impact of HPV vaccination in invasive disease remains unclear, and future studies are needed to evaluate its effect in a particularly heterogeneous group of patients, including those subjected to fertility-sparing procedures, those treated more radically with surgery and patients with advanced-stage disease receiving chemoradiotherapy. 

## 5. Recurrent Disease Challenges

Current recommendations suggest that patients with recurrent disease that have already received a full course of pelvic irradiation (including external beam radiation with or without brachytherapy) should refer to tertiary centers with particular expertise, as centralization and participation in clinical trials are encouraged. Pelvic exenteration as described by Brunschwig A. and laterally extended endopelvic resection may help achieve adequate disease remission in cases that have been previously irradiated; however, both operations are accompanied by significant morbidity. The latest evidence presented in the international literature related to persistent and recurrent cervical cancer suggests that approximately half of those patients are alive at 5 years [54,55,56]. In specialized centers, the operation is feasible even with minimally invasive surgery, with survival rates equivalent to those achieved with laparotomy [56]. Nevertheless, a significant matter of concern is the poor overall quality of life, which seems to be translated in unsatisfactory functional, social and symptom-related scores [57]. Certain patients seem to have a short post-recurrence survival, which is the result of negative prognostic factors, including the presence of pelvic lymph node metastases, early postoperative complications requiring surgical re-exploration, the need to perform infralevatoric resection of the tumor and the presence of positive resection margins [55,57]. Considering this, it becomes apparent that optimal preoperative assessment of these cases is of paramount importance in order to help increase survival rates and carefully select patients that will more likely benefit from the procedure. Unfortunately, modern imaging does not seem to be as accurate as is needed to help define cases at risk of having positive resection margins [58]; hence, further investigation is required through combined sharing of information between radiologists and surgeons.

A promising method for these patients is intraoperative radiation therapy. At least 14 studies have been published since the early 1990s referring to the feasibility and survival benefit of the technique in cases with cervical cancer [59]. Of these, eight studies involved exclusively cases with recurrent disease. Unfortunately, the used cohorts were particularly heterogeneous as the operations that were conducted varied from simple biopsy to total pelvic exenteration, therefore rendering impossible the estimation of the actual benefit of the technique in patients that can achieve nearly complete excision of the disease but in the presence of microscopically detectable positive margins. 

## 6. Conclusions

Current advances in surgical oncology necessitate the conduction of further studies as novel evidence from retrospective cohorts becomes available. While early-stage disease has been extensively investigated during the last decade, increasing the possibility of less radical approaches and extending the possibilities for fertility-sparing management, the surgical options in advanced-stage disease have several gray fields, and in the era of personalized medicine every effort must be made to help establish robust evidence that will guide patient counseling and increase the oncological safety of selected procedures. Relapsing disease in priorly irradiated tumors remains also poorly explored and, while international societies dictate the need for centralized handling and inclusion of these patients in clinical protocols, research is still avoided as the number of extended, out-of-the-box procedures gradually declines at the international level. While the advances in early-stage disease should continue, further efforts are necessary to determine if surgery may be an option for selected cases with advanced-stage and relapsing disease in order to help optimize survival outcomes of the vast majority of cervical cancer patients that present with advanced disease.

## Data Availability

Not applicable.
